# Warming and Wetting will continue over the Tibetan Plateau in the Shared Socioeconomic Pathways

**DOI:** 10.1371/journal.pone.0289589

**Published:** 2023-08-04

**Authors:** Hao Deng, Zhenming Ji

**Affiliations:** 1 School of Atmospheric Sciences, Sun Yat-sen University, Zhuhai, 519082, China; 2 Key Laboratory of Tropical Atmosphere‐Ocean System, Ministry of Education, Zhuhai, 519082, China; 3 Southern Marine Science and Engineering Guangdong Laboratory, Zhuhai, 519082, China; Universiti Sains Malaysia, MALAYSIA

## Abstract

We have used bias-corrected data from CMIP6 models to drive a regional climate model and project climate on the Tibetan Plateau (TP) in the 21st century. Changes in two background fields, namely, 2-meter air temperature and total precipitation, were analyzed. The results show that the WRF simulations capture the terrain effect that cannot be represented in low-resolution models. The simulation of temperature is better in summer than in winter, while the simulated precipitation is the opposite. By the end of the 21st century, the entire TP region experiences significant warming, with an average warming of 3°C and 7°C in the SSP245 and SSP585 scenarios, respectively. The western region shows a greater warming amplitude, with a maximum of more than 10°C in the SSP585 scenario. Most regions of the TP had significant increases in precipitation by the end of the 21st century, with precipitation increasing by 90 mm and 200 mm in the two scenarios, respectively. However, in the low-altitude areas of southeastern TP, total winter precipitation is significantly reduced in the SSP585 scenario. The strengthening of the East Asian summer monsoon and westerly disturbances collectively leads to a significant increase in precipitation within the TP region. By the end of the 21st century, the average annual precipitation in the TP is projected to reach approximately 600 millimeters.

## 1. Introduction

Covering over 2.5 million km^2^ in Asia, the Tibetan Plateau (TP) is a vast and unique geographical feature [1,2]. Known as the "Third Pole," it boasts massive ice fields and permafrost zones, making it the largest of its kind in the low and middle latitudes [3]. While the terrain of the TP is generally high in the west and low in the southeast, the area above 4000m makes up a significant 73% of the plateau. However, the Qaidam Basin, located in the northern part of the TP, as well as small portions of the eastern and southern parts, have lower altitudes, with the lowest point being only 46 meters [1]. The complex and distinctive geographical environment of the TP has a profound impact on regional and global climate. For example, simulations conducted by the CESM model demonstrate that the absence of the TP causes the average temperature of the Northern Hemisphere to drop by 4 degrees Celsius and become drier [4]. Additionally, the existence of the TP blocks the westerly wind, which in turn affects thermodynamic circulation in the Atlantic Ocean. Furthermore, the TP strengthens the intensity of the Asian summer monsoon [2,4].

Climate models, both global and regional, are widely employed to forecast future climate patterns and their extremes, such as background climate conditions in China [5,6], tropical cyclones [7,8], and other climatic anomalies [9]. Many studies have examined how global warming affects China, with a focus on near-surface temperatures and precipitation [5,6,10,11]. A recent investigation employed data from five models in CMIP5 to drive the RegCM4 numerical model and simulate changes in near-surface air temperature and precipitation over the Tibetan Plateau (TP) in the RCP4.5 scenario [12]. The simulation results suggest that winter temperatures in most parts of the TP will increase by over 3.5°C, and summer temperatures will increase by 2–3°C during the final two decades of the 21st century. Furthermore, winter and summer precipitation will increase by 5–25 mm, with a slightly more pronounced increase in winter. However, there is a lack of high-resolution regional numerical models that specifically investigate the TP. This study utilizes improved spatial resolution, more accurate driving field data, and multiple temperature rise scenarios to yield richer and more realistic simulation outcomes.

This paper is organized as follows: Section 2 provides a detailed description of the numerical models, specific parameter settings, observational data, and driving field data utilized in the study. Section 3 conducts a comprehensive comparative analysis of the model data and observational data during the historical period, providing insights into the accuracy and reliability of the model results. Section 4 analyzes the model results for the end term of the 21st century, examining the projected changes and potential impacts on the research subject. Finally, Section 5 summarizes and discusses the key findings, implications, and future research directions.

## 2. Model, data and methods

We conducted simulations using the WRF numerical model [13] for two periods: the past (1985–2014) and the future (2015–2100). The model uses the Lambert projection with a horizontal grid spacing of 20km and 41 vertical layers, covering the central and western regions of China, most of India, the western region of Southeast Asia, and the eastern region of West Asia. To ensure model stability and save time during the long simulation period, we perform re-initialization every 5 model years and set a spin-up time of half a year to obtain stable simulation results. The parameterization scheme is set up as follows: Purdue Lin scheme for Microphysics [14], Grell-Freitas scheme for Cumulus Parameterization [15], Unified Noah land-surface model for the land surface scheme, and RRTMG scheme for Longwave radiation and shortwave radiation.

We used the bias-corrected data from the CMIP6 dataset as the driving field, which was referred to as CMIP6bc [16]. The original data was corrected for bias using the ERA5 reanalysis data, and a non-linear trend correction was applied using 18 CMIP6 models [17]. This correction effectively removed disturbance and trends, leading to data that better reflects the actual climate conditions and shows significant improvement compared to the original data, particularly in terms of climate averages and extreme events. In addition, the multi-model ensemble (MME) approach significantly reduces the uncertainty of single-model estimates, thus enhancing the reliability of the results.

We provided a comparative analysis between the simulation results of WRF for the past period and two datasets: CN05.1 grid data and ERA5 reanalysis data. CN05.1 grid data is a high-resolution grid point dataset (0.25°×0.25°) based on observations from over 2,400 stations affiliated with the China Meteorological Administration [18]. As an observational dataset, it provides a more realistic representation of weather conditions compared to other datasets. Previous studies have shown that the precipitation field data of ERA5 in China is of higher quality than that of ERA-Interim [19]. ERA5 is based on a large amount of observational data and advanced meteorological models, and has a very high temporal and spatial resolution, making it a valuable reference dataset [20].

## 3. Results

### 3.1 Validation of historical simulation

**Fig 1:**
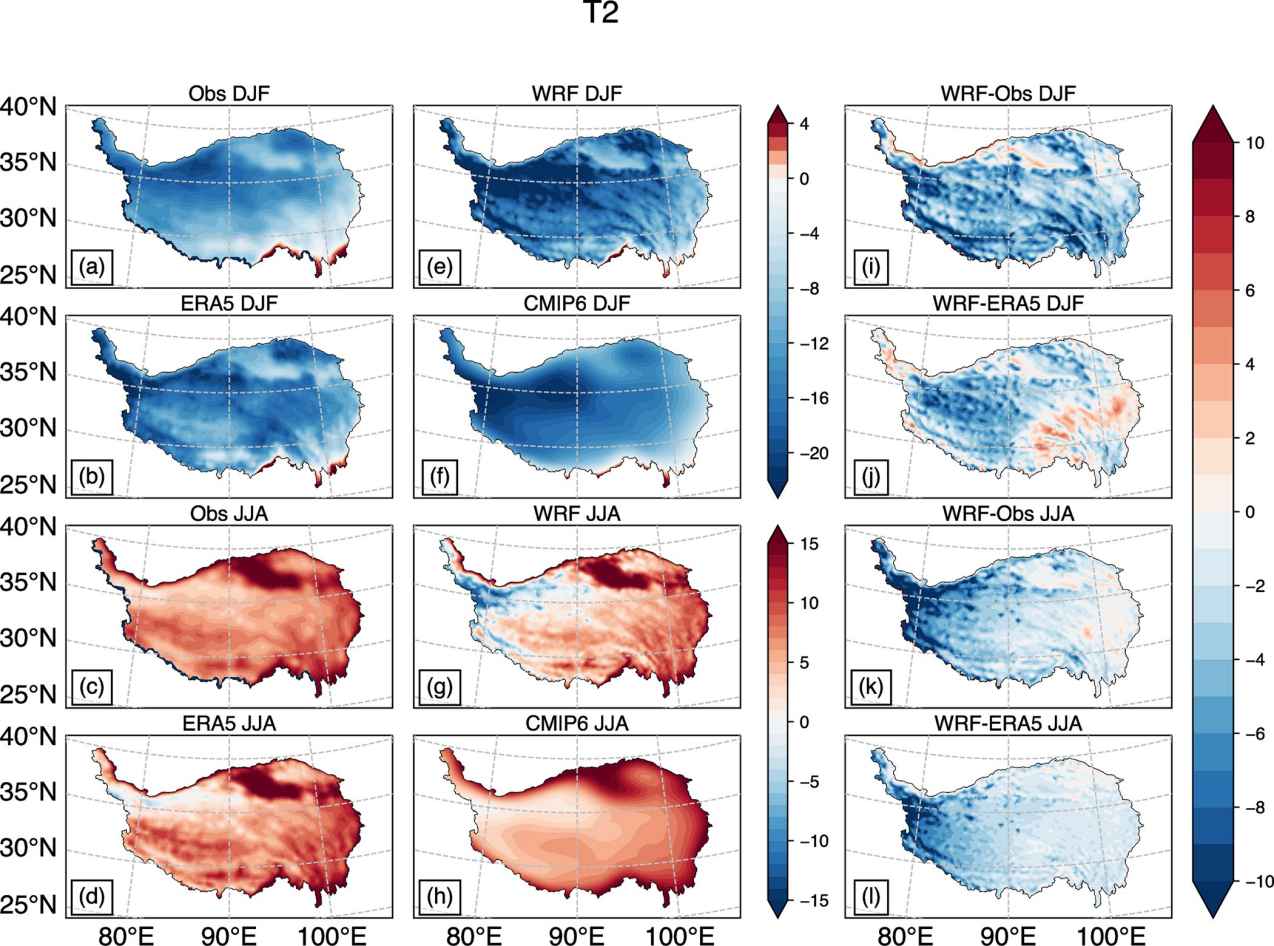
Distribution map of average temperature of air at 2m (°C) in winter and summer from 1985 to 2014: Observation in DJF (a), JJA(c); WRF simulation (e, g); ERA5 (b, d); The ensemble mean of CMIP6 18 numerical models (f, h); Difference between WRF and observation (i, k); Difference between WRF and ERA5 (j, l).

Fig 1 illustrates the distribution of mean air temperature at 2 meters in winter and summer from observational data, WRF simulation results, ERA5 and CMIP6 ensemble averages. The WRF simulation results are generally cooler than the observational data in winter (Fig 1E and 1I), especially in the western Kunlun Mountains, the western Gangdisi Mountains, and the high-altitude areas of the Hengduan Mountains, where temperatures can be as much as 10°C lower. Conversely, the WRF simulation results are slightly warmer in the southwestern side of the Qaidam Basin, with an amplitude below 4°C. These differences may be due to the lack of observation stations in these areas. There are only three observation stations near the west of the Gangdisi Mountains, and none in the west of the Kunlun Mountains. Although more observation stations are available at the junction of the Tarim Basin and the TP, the results interpolated in regions without surface observation stations may not reflect the actual temperature well (Fig 1A) [18,21].

ERA5 data showed similar results to WRF simulations in the above-mentioned mountain areas (Fig 1B). Moreover, Fig 1J indicates that the overall difference is smaller than (i) because ERA5 data absorbs more types of observation data, such as satellite data. This makes its data in areas where ground observation stations are lacking more realistic than the results obtained by interpolation. On the other hand, CMIP6 results exhibited the poorest performance (Fig 1F), failing to capture the terrain effects caused by mountains due to the low resolution of the global model. Note that there are two minimum centers in the northwest region of the TP in (f), which may be one of the reasons why the WRF simulation results at this location are relatively low.

The simulation results from WRF for summer are still cooler, but they are better compared to the winter simulation results. In most parts of the central and eastern TP, the temperature difference does not exceed 3°C (Fig 1K and 1L). WRF performed well in simulating high temperatures in the Qaidam Basin, the northern Gangdisi Mountains, and other low-elevation areas (Fig 1C, 1D and 1G). In the northwestern part of the TP, the simulated results are slightly cooler. Although the spatial distribution of the CMIP6 data is similar to the other three results (e.g., the temperature maximum areas located in the two regions mentioned above), the resolution is too coarse to reflect detailed information (Fig 1H).

**Fig 2:**
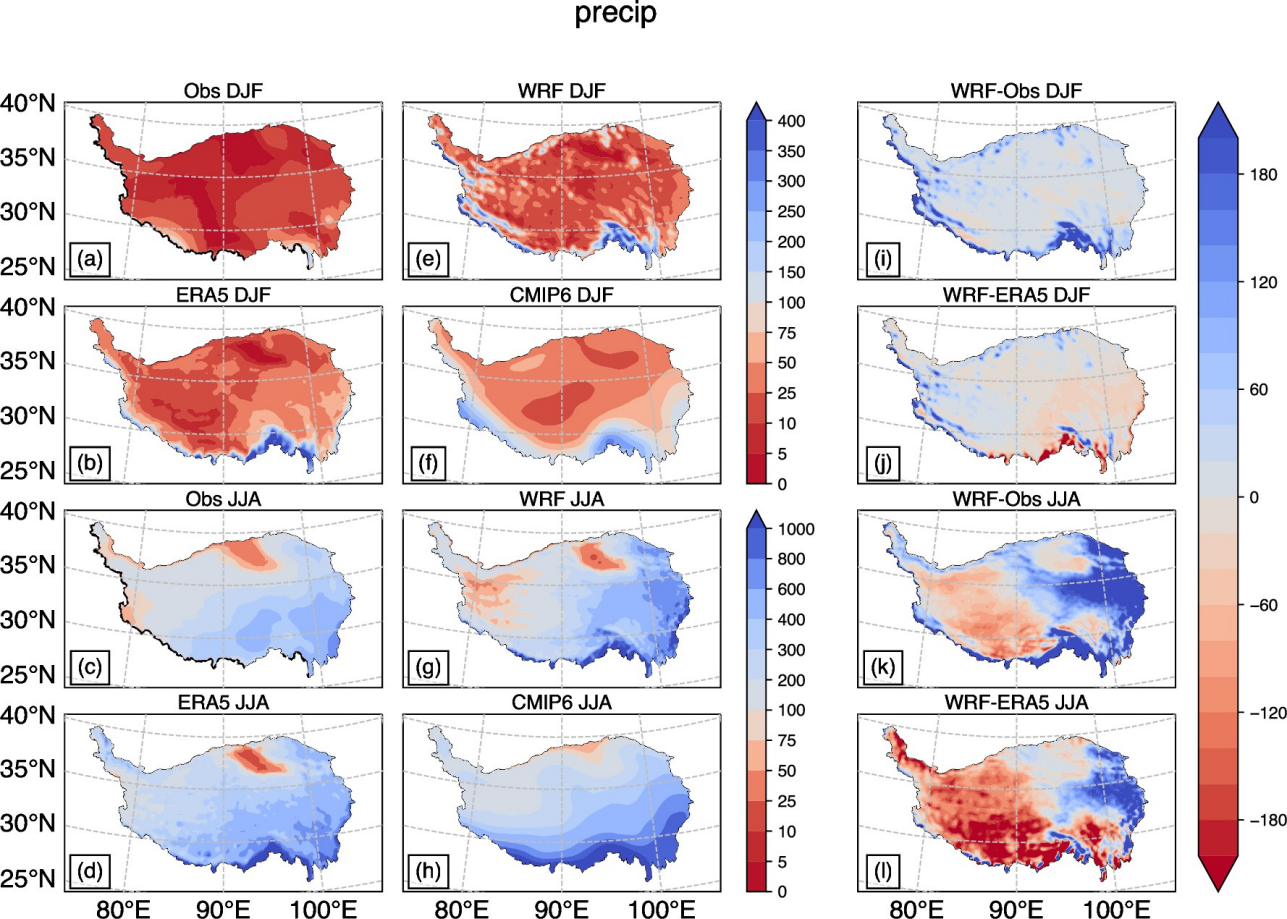
Distribution map of total precipitation (mm) in winter and summer from 1985 to 2014: Observation in DJF (a), JJA(c); WRF simulation (e, g); ERA5 (b, d); The ensemble mean of CMIP6 18 numerical models (f, h); Difference between WRF and observation (i, k); Difference between WRF and ERA5 (j, l).

Fig 2 depicts the spatial distribution of total precipitation over the TP during winter and summer from 1985 to 2014, based on four types of data. In winter, all four datasets show a similar spatial pattern, with little precipitation observed in most areas except for the southern margin of the plateau. However, Fig 2A reveals that the observed precipitation data differ from the near-surface air temperature and hardly reflect the terrain effect. Interestingly, an area with an altitude less than 1500m on the south side of the plateau exhibits more than 100 mm of total precipitation for WRF, ERA5, and CMIP6 data, and the maximum total precipitation can reach 300 mm (Fig 2B, 2E and 2F). This finding is supported by some RCMs and GCMs simulation results [5,12]. The high precipitation in this area can be attributed to the southern winter westerlies moving from west to east along the southern side of the plateau and getting obstructed by the Hengduan Mountains, leading to the uplift of the airflow and subsequent precipitation [2,22]. The lack of surface observation stations in this area may explain why observed data do not demonstrate significant precipitation here. Results obtained by interpolation cannot fully capture the precipitation caused by drastic topographical changes. Moreover, the spatial distribution of total precipitation near the Qaidam Basin differs significantly from that of WRF, ERA5, and CMIP6.

During the summer season, similar to the winter observations, there is little precipitation in the low-altitude areas of the southern Tibetan Plateau as shown in Fig 2C. However, the Qaidam Basin shows a better representation of areas with less precipitation, with similar shapes and extents to the WRF and ERA5 data (Fig 2D and 2G). On the other hand, the ensemble average data of CMIP6 fails to reflect the range of dry areas in the Qaidam Basin, as the northern part of the Tibetan Plateau is relatively humid (Fig 2H). Additionally, Fig 2G shows a total precipitation of 50 to 75 mm less than the other three datasets on the west side of the Plateau. However, based on Global Precipitation Measurement (GPM) data and previous studies, a horizontal band in the northwest of the Tibetan Plateau has a total annual precipitation of less than 100 mm [22,23]. Using GPM as the standard, the data from observations, ERA5, and CMIP6 are relatively large, while the WRF simulation results are more consistent with GPM.

In terms of the difference in precipitation, the WRF simulation results for total precipitation in winter are more accurate than those in summer, in contrast to the temperature results, largely due to the significantly lower total precipitation in winter compared to summer. However, when considering the deviation percentage, the simulation results in winter are not as satisfactory. The simulation results in many areas of the TP exceed the observed data by more than twice. The difference between WRF simulated data and observations is less than 40 mm in most areas of the Tibetan Plateau during winter, except for the low-altitude regions in the western Gangdisi Mountains and southern TP (Fig 2I and 2J). However, during summer, WRF tends to underestimate total precipitation in the west and south, and overestimate it in the east of the TP (Fig 2K and 2L). The possible reason for the underestimation of summer precipitation in the southern part of the TP by WRF is that the model simulates less water vapor transport than observed data [24]. On the other hand, the overestimation in the eastern region may be due to higher surface temperatures (Fig 1K), leading to stronger local convection and consequently more precipitation. There are also studies that have examined the discrepancies between RCM results and observed precipitation data. In these studies, the deviations in most regions range from 10 to 50 millimeters in winter and from 50 to 200 millimeters in summer [12,24,25]. The deviations in this article fall within these ranges, with some regions showing slightly better results compared to previous studies.

The deviations in WRF simulation results for surface temperature and precipitation may also manifest in future simulations. The temperature simulation results for future periods may generally exhibit a bias towards colder conditions compared to the actual scenario. As for precipitation, the simulation results indicate a tendency towards drier conditions in the western and southern parts of the TP, while the eastern region tends to be wetter.

**Fig 3:**
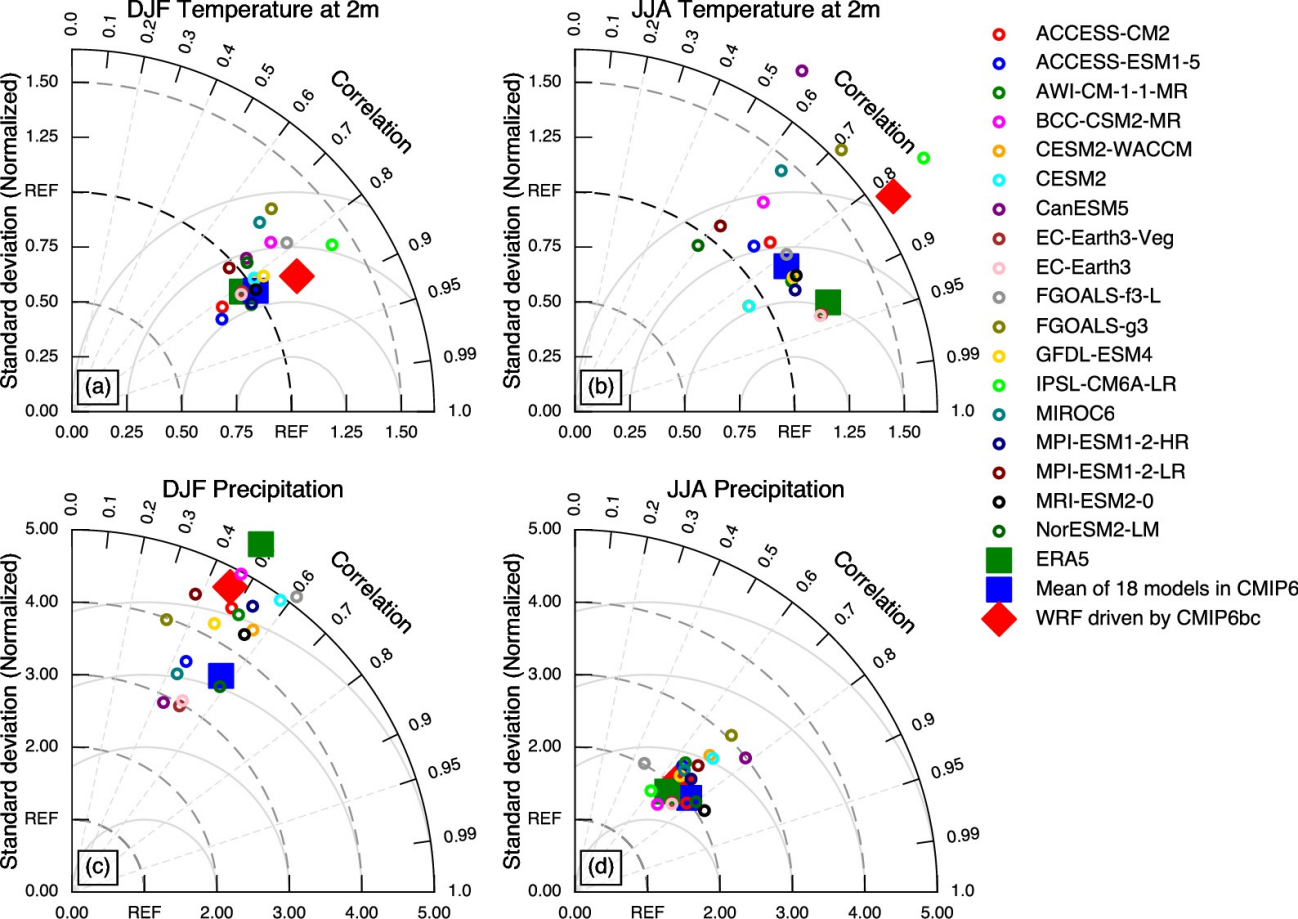
Taylor diagrams for the spatial distributions of near-surface temperature and precipitation in winter and summer from 1985 to 2014. The observational data were used as the reference. All data has passed the Pearson correlation coefficient test at a significance level of 0.05.

We validated the accuracy of the simulation results using Taylor diagrams. Fig 3 presents the verification results for near-surface temperature and precipitation during winter and summer. It can be observed that WRF exhibits a larger negative bias in simulating near-surface temperature in the northwest region (Fig 1E and 1G), resulting in a higher spatial standard deviation compared to ERA5 and the CMIP6 MME (Fig 3A and 3B). Nevertheless, the spatial correlation coefficient of near-surface temperature simulation results slightly outperforms the CMIP6 MME during summer and surpasses both the CMIP6 MME and ERA5 during winter.

The correlation coefficients for winter precipitation between ERA5, the CMIP6 MME, and WRF simulation results are relatively low (Fig 3C). This is mainly because all three show a significant overestimation of precipitation in the southern region of the TP compared to the observed data (Fig 2B, 2E and 2F). WRF exhibits a pronounced overestimation of precipitation in the mountainous regions in western and northern TP, resulting in the lowest correlation coefficient among the three datasets. Due to the averaging of multiple models, the CMIP6 MME results have a smoother spatial distribution, leading to a smaller standard deviation compared to WRF and ERA5. ERA5 significantly overestimates precipitation in the low-altitude southeastern region of the TP, resulting in a much higher standard deviation than the WRF simulation results. In summer, the performance of the three datasets is quite similar. WRF simulation results show a balanced performance, while ERA5 underestimates precipitation in the Qaidam Basin, and CMIP6 exhibits a significant overestimation in large areas of the southern TP. This leads to slightly poorer performance in terms of standard deviation for ERA5 and CMIP6 compared to the WRF simulation results.

### 3.2 Future projection and trends

In this section, we analyzed the near-surface air temperature and total precipitation from two perspectives: spatial distribution differences and changes in their spatial averages over time.

**Fig 4:**
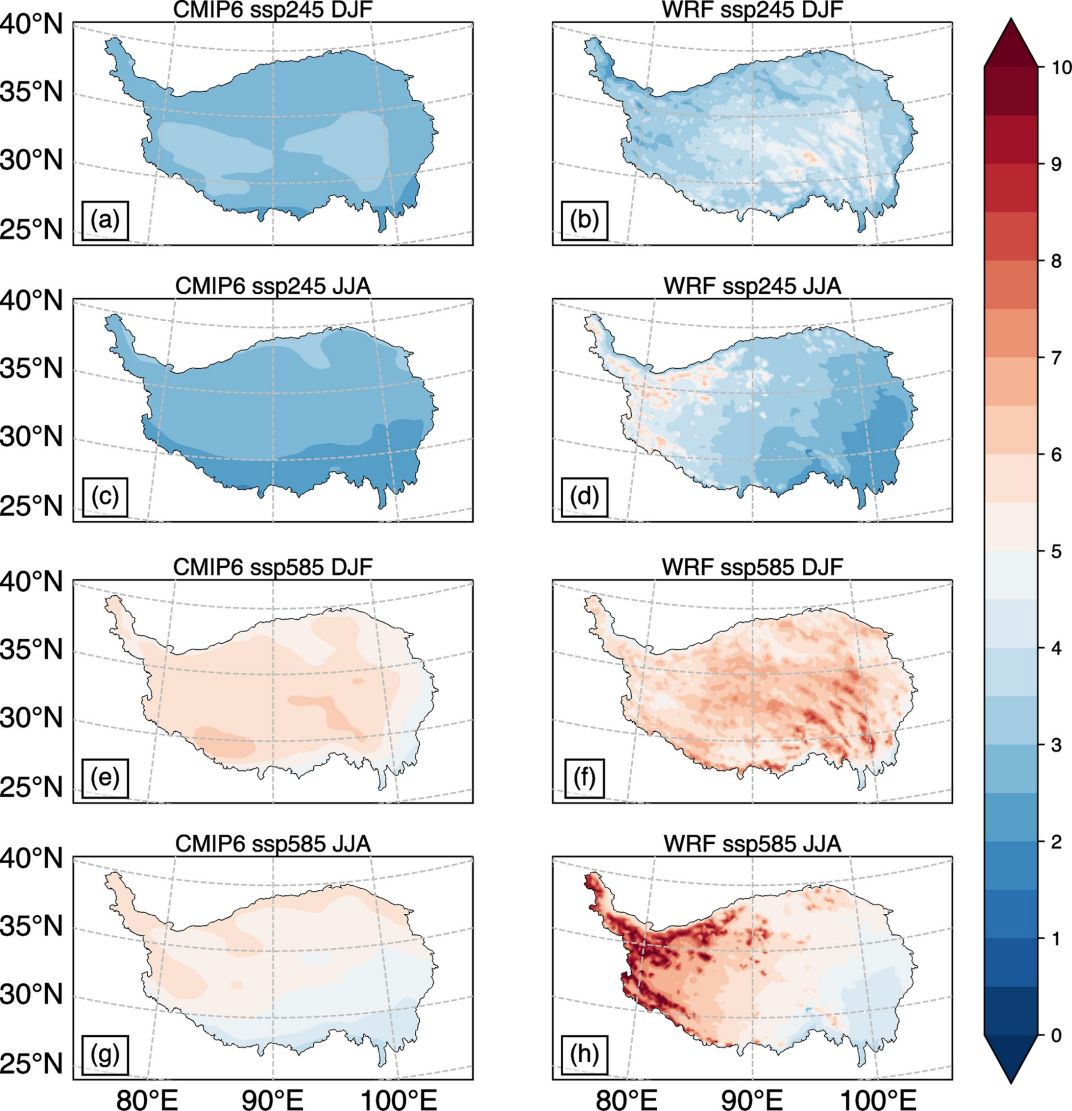
Mean temperature changes (units: °C) in 2071–2100 relative to 1985–2014: CMIP6 ensemble mean in DJF(a), JJA(c), WRF simulation results in DJF(b), JJA(d) under the SSP245 temperature scenario; CMIP6 ensemble mean in DJF(e), JJA(g), WRF simulation results in DJF(f), JJA(h) under the SSP585 temperature scenario. All 8 graphs in the whole region pass the student’s t-test with 95% confidence interval.

Fig 4 illustrates the simulation results of near-ground temperature at the end of the 21st century (2071–2100) from CMIP6 ensemble mean and WRF. In the SSP245 and SSP585 scenarios, the temperature increases in most parts of the TP range from 2 to 6°C and 4 to 8°C, respectively. WRF simulation results show a 1–3°C higher temperature than CMIP6 (Fig 4A, 4C, 4E and 4G). The low average temperature of CMIP6 can be attributed to the considerable simulation results of different models, particularly in some place in the west of TP, where some models indicate a temperature decrease over time, which is contrary to the multi-model ensemble mean trend [26–28]. [29] pointed out that the cooling observed in the western TP is largely associated with deforestation and soil degradation, within the context of global warming. However, when considering the average surface temperature across the entire TP, these models still show an increasing trend over time (Fig 6) [27].

With higher resolution grid for simulation, WRF results present richer details than CMIP6. The temperature increase is higher in the Hengduan Mountains than in other areas during winter, and the Himalayas, the Gangdisi Mountains, the Kunlun Mountains, and the northwest of the northern TP during summer under the SSP245 and SSP585 scenarios (Fig 4B, 4F, 4D and 4H). This spatial distribution of temperature increase in the eastern part of the TP during winter and the western part during summer is also evident in the historical observation data [30].

The areas with higher temperature increase are mainly in high altitude areas, which is consistent with the Snow/ice-Albedo’s positive feedback mechanism [27]. In mountainous areas, the substantial amount of snow, when combined with global warming, triggers the positive feedback mechanism, leading to snow melting and resulting in a higher temperature in the mountainous region than other areas. This phenomenon eventually leads to the elevation dependent warming (EDW) phenomenon [27,31].

**Fig 5:**
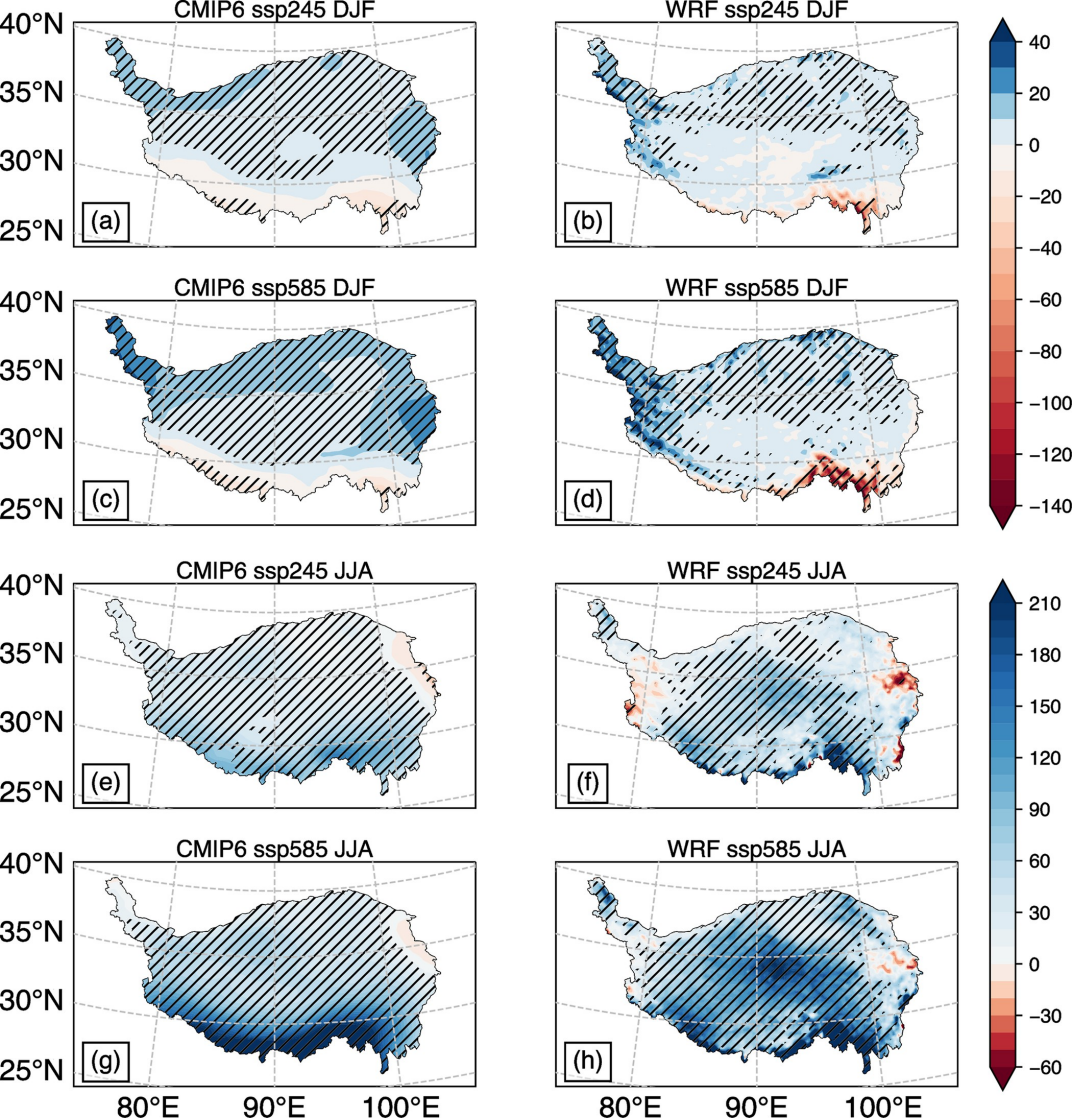
Total precipitation changes (units: mm/season) in 2071–2100 relative to 1985–2014: CMIP6 ensemble mean in DJF(a), JJA(e), WRF simulation results in DJF(b), JJA(f) under the SSP245 temperature scenario; CMIP6 ensemble mean in DJF(c), JJA(g), WRF simulation results in DJF(d), JJA(h) under the SSP585 temperature scenario. The areas covered by the slashes pass the student’s t-test with 95% confidence interval.

Fig 5 illustrates the distribution of total precipitation variation in winter and summer under different temperature rise scenarios at the end of the 21st century. As shown in the figure, total precipitation in most regions of the TP has increased to varying degrees. Specifically, there is a decrease in precipitation mainly in the southern part of the TP during winter, while in summer, precipitation decreases mainly in the eastern and western parts of the plateau.

In winter, precipitation shows relatively large changes in percentage, as it is generally less than 50mm in most areas of the TP (Fig 2A, 2B, 2E and 2F). While the variation in precipitation is within the range of ±10mm in most regions of the TP, the ensemble average of CMIP6 shows that precipitation significantly increases in the northwest and eastern parts of the TP in the two scenarios (Fig 5A and 5C). Conversely, precipitation significantly decreases in the southwest and southeast regions. The WRF simulation indicates that the areas with a large increase in precipitation are mainly in the Gangdisi Mountains and the northwest of the TP, where elevations are higher. Conversely, precipitation decreases significantly in the southeast where the altitudes are less than 1000 m. With higher temperature rise, the area of significant reduction becomes larger and the reduction amplitude becomes greater (Fig 5B and 5D).

Different studies have reported different conclusions on whether total winter precipitation in this region will increase or decrease by the end of the 21st century. For instance, Fu, Gao (12) used data from five GCMs in CMIP5 to drive RegCM4 for simulation, and the results show that in the RCP4.5 scenario, the total winter precipitation in the region increased by 0 to 25 mm and passed the significance test at the 95% confidence level. But the ensemble mean of the driving fields showed a decrease of 0.5 mm in some areas. Chen and Gao (5), who used CSIRO 3.6.0 to drive the RegCM4 model, showed that the total precipitation in the region decreased by 0.25% in the RCP4.5 and RCP8.5 scenarios, with a smaller decrease in total precipitation in the RCP8.5 scenario. Similarly, Lu, Huang (11) used ERA-Interim and CMIP5 as driving field data for simulation, and the results showed that total precipitation increased by 0–15 mm in the RCP4.5 and decreased by 0–15 mm in the RCP8.5.

The significant differences among the results of different studies are primarily due to the use of different driving field data. The aforementioned studies utilized data from various models in CMIP5, with a relatively limited number of models used in each study. It has been noted that there is considerable variation in the accuracy of precipitation data among different models in CMIP5 [32], leading to significant discrepancies in the output of regional models. However, compared to CMIP5, the precipitation data in CMIP6 show a significant improvement in average accuracy. After further bias correction, the 850hPa wind field near the TP becomes closer to reality [16]. Wind plays a crucial role in the transport of water vapor, and improving the wind data in the driving fields contributes to obtaining more accurate prediction results.

In summer, the total precipitation in most areas of the TP increases significantly, and the increase is generally larger than in winter. Both the CMIP6 and WRF simulation results show that the southern part of the TP experiences the largest increase in precipitation, with a maximum increase of more than 200 mm (Fig 5E, 5F, 5G and 5H). This is because the summer monsoon from the Indian Ocean brings abundant water vapor and is forced to rise by the steep terrain when it reaches the southern part of the TP. Therefore, the region with heavy precipitation should have a strong terrain effect, and the distribution of precipitation in Fig 5H is more reasonable than that in Fig 5G. Besides the southern TP, the WRF simulation results suggest that there is also a large-value area of total precipitation change in the central TP. Additionally, the total precipitation decreases in the west and east of the TP, but only a small area passes the significance test (Fig 5F and 5H).

**Fig 6:**
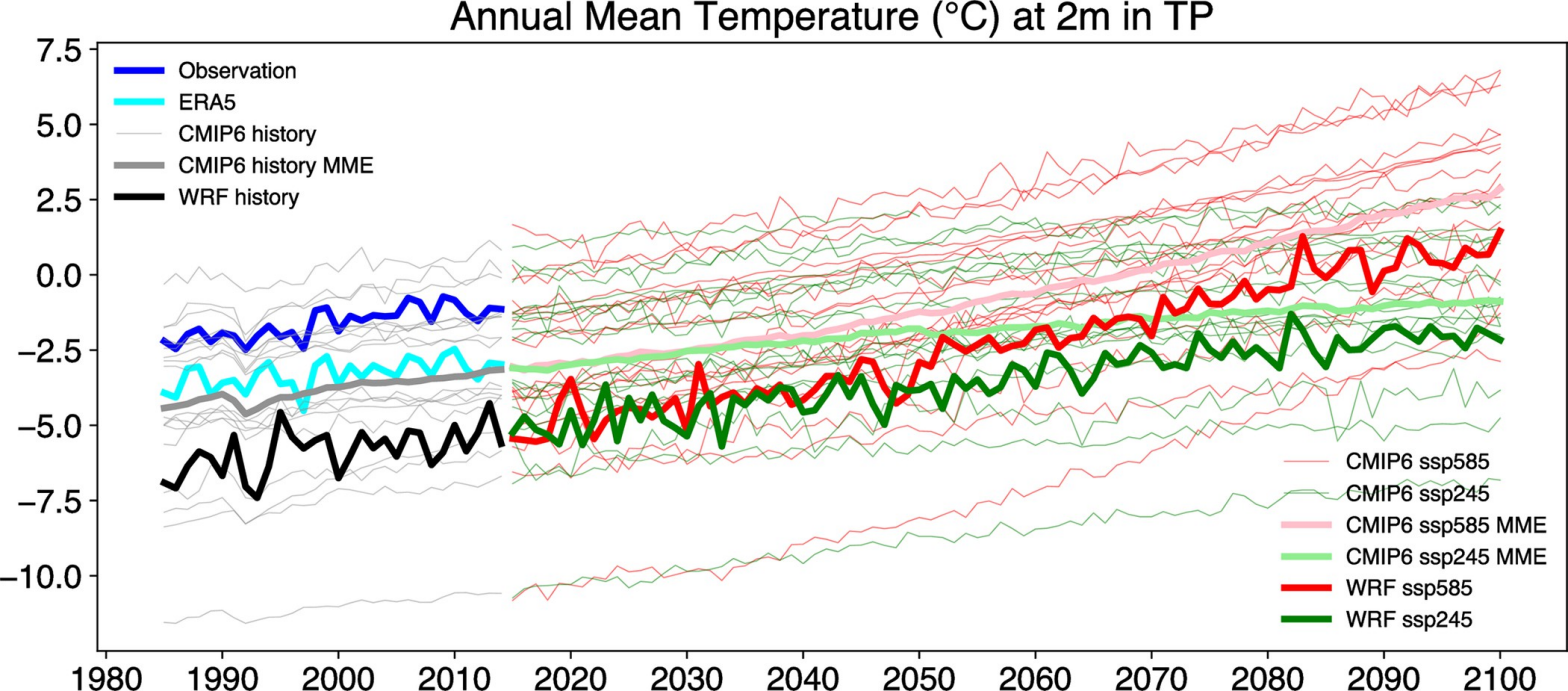
Annual mean temperature at 2 meters in the TP from 1985 to 2100 based on different data sources and warming scenarios. All data has passed the Pearson correlation coefficient test at a significance level of 0.05.

Fig 6 presents the annual mean temperature at 2 meters in the Tibetan Plateau from 1980 to 2100 under different data sources and scenarios. The WRF simulations exhibit a similar trend to the CMIP6 simulations, but with greater interannual variability than most CMIP6 models. In the historical period, the WRF-simulated annual mean temperature in the Tibetan Plateau has increased by approximately 1.5°C. Although the temperature values differ from the observed data, the rate of temperature increase is comparable to that of the observed data. The results of CMIP6 in the SSP585 scenario, the annual mean temperature in the Tibetan Plateau is projected to increase rapidly by around 7°C by 2100, while the SSP245 scenario predicts a moderate warming of approximately 3°C by 2100. It is worth noting that in the SSP585 scenario, the annual mean temperature is expected to remain above 0°C after 2090.

The reason for the significant differences among the observational data, ERA5, and WRF simulation results is the large discrepancies in data in mountainous regions, particularly in the northwestern part of the TP (Fig 1I, 1J, 1K and 1L). The differences between WRF and the actual conditions may also arise in future simulations. However, due to the snow/ice-albedo’s positive feedback mechanism mentioned above, this positive feedback process is likely to be stronger in the WRF simulation compared to the actual conditions. This could result in a reduction of the bias between simulated surface temperature and the actual conditions by the end of the 21st century, with the degree of reduction being more significant as the magnitude of warming increases.

Based on the evidence presented in Figs 4 and 6, it can be concluded that the TP is experiencing a significant warming trend, which is expected to continue and intensify in the future in the different emission scenarios. While WRF captures more details and uncertainties than CMIP6, both models agree on the direction and magnitude of the warming. The figures also suggest that the choice of emission pathway will have a significant impact on future climate change in the TP. Thus, reducing greenhouse gas emissions can help mitigate some of the warming effects.

**Fig 7:**
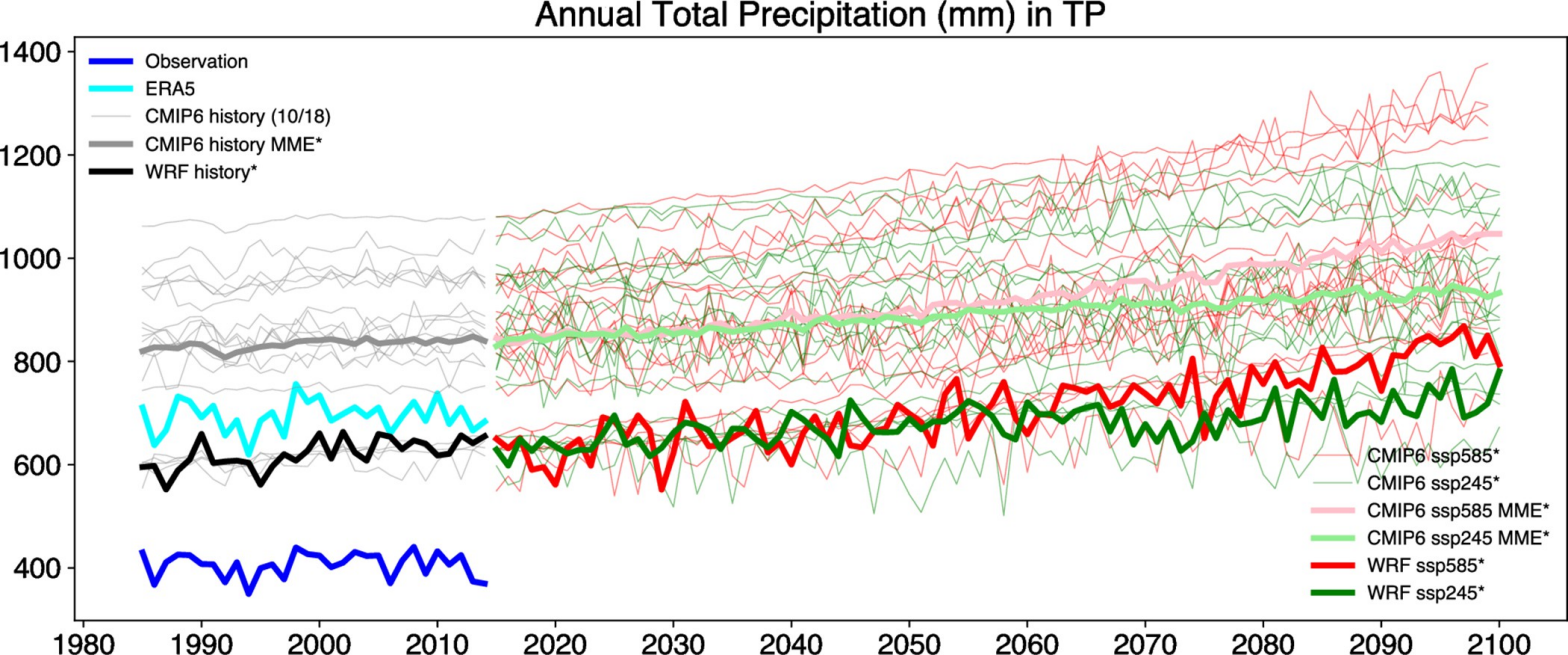
Annual total precipitation in TP from 1985 to 2100 based on different data sources and warming scenarios. The asterisk denotes passing the Pearson correlation coefficient test at a significance level of 0.05. In the CMIP6 historical experiment, out of 18 models, 10 have passed the significance test.

Fig 7 illustrates the annual total precipitation in TP from 1980 to 2100. It is evident from the observation data that the precipitation amount is significantly lower than that of ERA5 and WRF simulations. The first reason is the significant overestimation in the eastern part of the TP during summer (Fig 2K). The second reason is that the observation data for precipitation in the low altitude regions of the southern TP is much lower than the results of ERA5 and WRF simulations, as shown in Fig 2 [28]. The former’s maximum annual total precipitation in this area is only around 1000 mm, while ERA5 and WRF can exceed 3000 mm, with most areas having an annual total precipitation of over 2000 mm. This significant difference in precipitation in the area leads to the marked difference in the average precipitation amount. Zhu and Yang [28] suggest that the poor performance of the model simulations is the reason for such a large difference in precipitation in the region. As discussed earlier, it has been pointed out that this area lacks observation stations, and we believe that this is also one of the main reasons for the significant differences between the observation products and simulation results.

During the historical period, no significant upward trend was observed in the total precipitation data and ERA5. Additionally, nearly half of the 18 models in CMIP6 also showed no significant upward trend during this period. However, in the future period, both the 18 models in CMIP6 and WRF simulation results exhibit a significant upward trend. The southern and central regions of the TP are primarily influenced by the East Asian summer monsoon (EASM). He, Wang [33] conducted a study on the spatial distribution of latent heat in the TP and its surrounding areas. The results revealed a significant increase in latent heat with temperature rise in the southern and central parts of the plateau, as well as in most of South Asia under the RCP8.5 scenario. This increase in latent heat can trigger low-level anomalous cyclonic circulation around the plateau, thereby enhancing the EASM and bringing about increased precipitation. During winter, the northwestern region of the TP is influenced by westerly disturbances (WDs). These disturbances transport moisture from the Atlantic and Mediterranean regions, leading to precipitation formation in the northwestern region of the plateau [34]. A Study have indicated a significant enhancement of the upper-level jet stream under the RCP4.5 and RCP8.5 scenarios, resulting in increased precipitation in the northwestern region of the TP [35], as also observed in Fig 5A–5D. The strengthening of both the EASM and WDs contributes to a significant increase in precipitation over the entire TP region. According to the regression analysis of the WRF simulation results, the annual total precipitation is projected to increase by about 200 mm over 85 years in the SSP585 scenario and by about 90 mm over the same period in the SSP245 scenario. Before 2060, there is little difference in precipitation between the two scenarios. However, from 2066 to 2074, there is a noticeable fluctuation and decrease in precipitation in the SSP245 scenario, and thereafter, the precipitation in the SSP245 scenario remains consistently lower than that of the SSP585 scenario. Taking into account the differences between WRF simulation results and observational data during the historical period, the actual annual total precipitation over the TP is estimated to be around 600 millimeters by the end of the 21st century.

## 4. Conclusion and discussion

In this study, we utilized bias-corrected CMIP6 ensemble data as the driving field to achieve more precise outcomes. To conduct the downscaled simulations of the TP from 1985 to 2100, we used the WRF regional model with a 20 km horizontal resolution. Our primary focus was to analyze the average near-surface air temperature and total seasonal precipitation fields from 1985 to 2014. Additionally, we also investigated the difference fields between the average fields of the years 2071 to 2100 and those of the previously mentioned period.

Our findings indicate that the spatial distribution patterns of the WRF simulations during the historical period were consistent with the observation and ERA5 reanalysis data. However, the WRF temperature simulations tended to be biased towards colder temperatures, which could be attributed to two factors. Firstly, the driving field exhibited a colder bias, particularly in the northwest region of the TP. Secondly, the scarcity of observational stations in the complex terrain of the TP, particularly at higher altitudes, may result in a larger deviation between the WRF simulations and the observed interpolated fields [12]. The WRF total precipitation simulations exhibited varying biases in different regions of the TP. Additionally, the WRF temperature simulations showed better results in summer than in winter, while the total precipitation simulations were more accurate in winter than in summer.

The WRF simulations of near-surface temperature and total precipitation at the end of the 21st century displayed a strong terrain effect, with higher warming in the eastern mountainous regions during winter and in the western mountainous regions during summer. Under the SSP585 warming scenario, the warming could exceed 10°C, while the average temperature increase was around 7°C. These findings suggest that the TP has an amplification effect similar to that of the North and South Poles [36,37]. Additionally, the WRF simulation results reveal that the low-altitude areas in the southeastern TP may experience decreased precipitation in winter and increased precipitation in summer at the end of the 21st century. The increase in summer precipitation is likely due to abundant water vapor transported by the summer monsoon. However, different simulation models yield varying results for winter precipitation in this region, and no consistent conclusions have been drawn to date. Consequently, further research in this area is needed, utilizing richer data and more model simulations to arrive at more credible and detailed conclusions.

The underestimation of winter mountain temperatures and the overestimation of summer precipitation in the eastern and southern regions are significant factors contributing to the overall biases in temperature and precipitation. To improve these biases, we can consider updating surface type data and topographic data, improving land surface process schemes, and using more appropriate parameterization schemes for convection. It is also crucial to integrate observational data from multiple sources for a more accurate assessment. The observational station coverage in the southwestern part of the TP is relatively sparse in the CN05.1 dataset. However, in recent years, new observational stations have been established, including six stations located north of the Himalayas [38]. Obtaining data from more observational stations would be beneficial for creating more accurate gridded observational datasets and for evaluating global and regional model results.

This article focused on the analysis of two background field variables, near-surface air temperature, and total precipitation. However, in the future, we plan to conduct more comprehensive research on other aspects, including extreme weather events, other weather elements, and the interactions between weather elements and anthropogenic aerosols. Chemical processes also play a crucial role in weather and climate in TP [39], but this study did not incorporate them. To improve climate predictions, incorporating chemical processes into regional model simulations is essential, and it’s also one of the important directions for our future work.
